# Supplementary material for: Association between oral microbiome and seven types of cancers in East Asian population: a two-sample Mendelian randomization analysis
Source: Front Mol Biosci. 2023 Nov 21;10:1327893. doi: 10.3389/fmolb.2023.1327893 (PMC10702768; doi:10.3389/fmolb.2023.1327893)
Supplement: Supplementary file 1 [file DataSheet1.ZIP › Supplementary Materials/MR plots of saliva/saliva═╝/Pancreatic cancer/pheno.133_to_pancreatic cancer_leave_one_out.pdf]

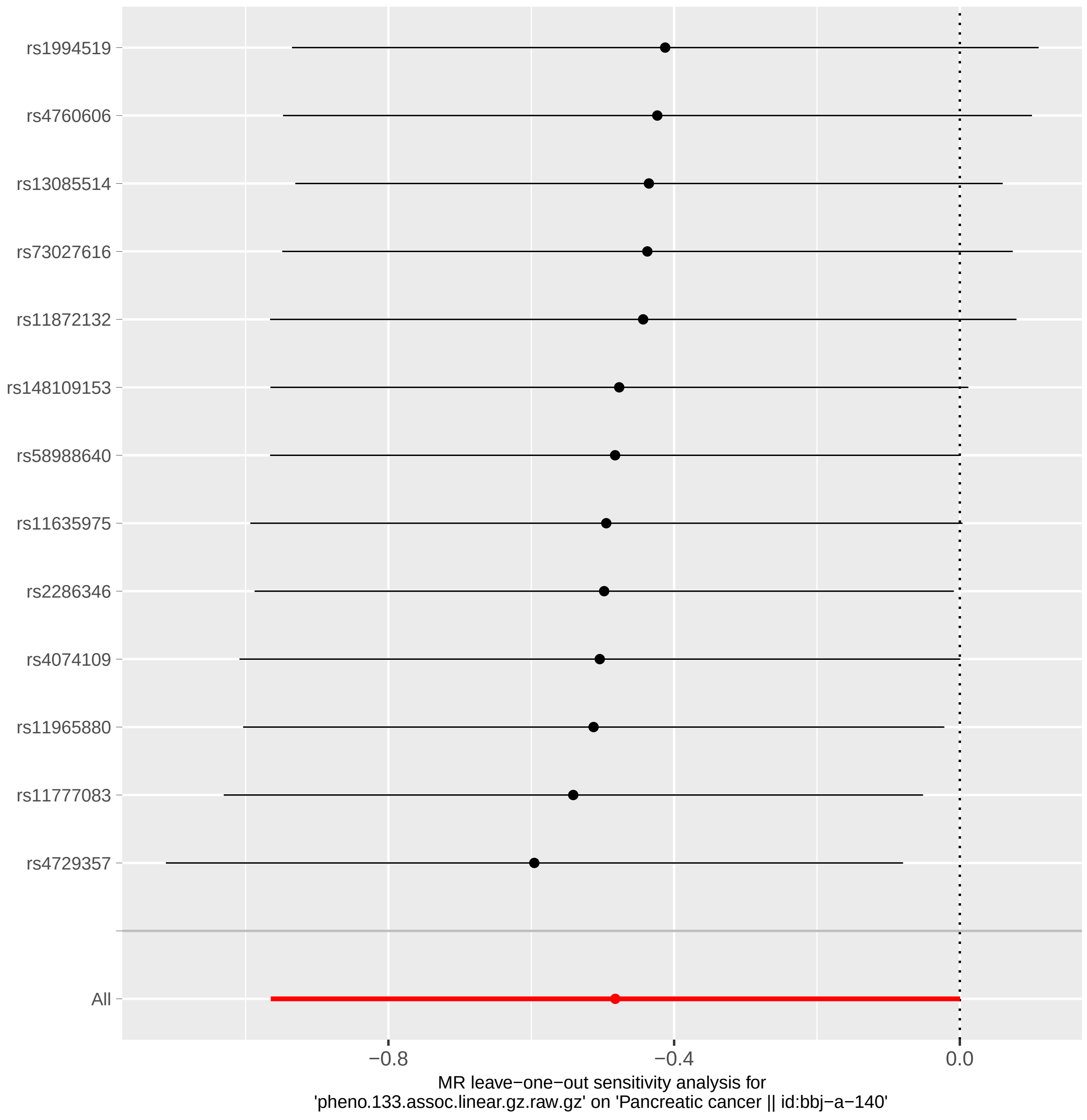
rs1994519
rs4760606
rs13085514
rs73027616
rs11872132
rs148109153
rs58988640
rs11635975
rs2286346
rs4074109
rs11965880
rs11777083
rs4729357
All
−0.8
−0.4
0.0
MR leave−one−out sensitivity analysis for
'pheno.133.assoc.linear.gz.raw.gz' on 'Pancreatic cancer || id:bbj−a−140'
